# Working Memory Decline after Age 60 Negatively Impacts Medication Adherence

**DOI:** 10.1093/geroni/igaf122.4049

**Published:** 2025-12-31

**Authors:** Candace Ryan, Cheryl Dahle, Debra Schutte, Ana Daugherty

**Affiliations:** Wayne State University, Detroit, Michigan, United States; Wayne State University, Detroit, Michigan, United States; Wayne State University, Detroit, Michigan, United States; Institute of Gerontology, Wayne State University, Detroit, Michigan, United States

## Abstract

Medication adherence is critical for chronic disease management. Working memory is a putative cognitive process necessary for successful adherence. Working memory is known to decline with age. Working memory processes are hypothesized to support successful adherence, which is vulnerable to age-related decline. Poor understanding of cognitive correlates to adherence among community-dwelling older adults impedes effective strategies to reduce late-life health disparities. A longitudinal analysis tested the association of working memory decline with future medication adherence in 117 community-dwelling participants of the Detroit Brain Aging Study (73.3% female, 84.3% White), mean age = 70.49 (SD = 12.48). Working memory was assessed on three measurement occasions (2-year delays), and medication adherence was reported at a follow-up 14–22 years after baseline. In binomial logistic regression, the interaction age × change in working memory predicted the likelihood of medication adherence (b = 0.72, p = 0.02). In the oldest age group >80 years, greater working memory declines predicted lesser likelihood of reporting medication adherence in the future, this was attenuated at younger ages, with a negligible effect at 60 years. Concurrent age, delay since baseline, and change in processing speed were not significant (all p’s > 0.7), nor was the cumulative model (p = .25; R2 = 0.11). In this sample, working memory declines had a modest association with future medication adherence behavior, with the effect most prominent among adults over age 60 years. Future studies are needed to determine additional factors, beyond cognitive decline, that contribute to medication adherence.

